# Mehr Evidenzbasierung in Prävention und Gesundheitsförderung: Kriterien für evidenzbasierte Maßnahmen und notwendige organisationale Rahmenbedingungen und Kapazitäten

**DOI:** 10.1007/s00103-021-03320-1

**Published:** 2021-04-21

**Authors:** Freia De Bock, Eva Rehfuess

**Affiliations:** 1 Abteilung 2 „Effektivität und Effizienz gesundheitliche Aufklärung“, Bundeszentrale für gesundheitliche Aufklärung (BZgA), Maarweg 149–161, 50825 Köln, Deutschland; 2 Institut für medizinische Informationsverarbeitung, Biometrie und Epidemiologie, Ludwig-Maximilians-Universität, München, Deutschland; 3 Pettenkofer School of Public Health, München, Deutschland

**Keywords:** Capacity Building, Wirksamkeit, Komplexe Interventionen, Systemperspektive, Praxis, Capacity building, Effectiveness, Complex interventions, Systems perspective, Practice

## Abstract

Für die Umsetzung des Präventionsgesetzes in einem sich entwickelnden System Prävention und Gesundheitsförderung (PGF) ist die Anforderung der Evidenzbasierung formuliert. Vor diesem Hintergrund stellt sich die Frage, welche Schritte, Prozesse und Vorgehensweisen in diesem System benötigt werden, um der Anforderung zunehmend gerecht zu werden. Dieser Übersichtsartikel diskutiert für Deutschland, wie evidenzbasierte Maßnahmen in der Praxis operationalisiert werden können und welche organisationalen Rahmenbedingungen und Kapazitäten für ein evidenzbasiertes Handeln von AkteurInnen notwendig sind.

Aufbauend auf internationalen wissenschaftlichen Erkenntnissen und dem Memorandum Evidenzbasierte Prävention und Gesundheitsförderung der Bundeszentrale für gesundheitliche Aufklärung (BZgA) wird zunächst das Verständnis von evidenzbasierten Maßnahmen erläutert und im Weiteren werden Elemente zur Umsetzung von mehr Evidenzbasierung skizziert.

Neben der transparenten und einheitlichen Darstellung in Datenbanken und Empfehlungen ist es notwendig, bei EntscheidungsträgerInnen in Praxis und Politik ein gemeinsames Verständnis von evidenzbasierten Interventionen und von Anforderungen für eine Evaluation, die Evidenzbasierung sichert, zu schaffen. Darüber hinaus kann evidenzbasiertes Handeln von EntscheidungsträgerInnen gefördert werden durch Wertschätzung gegenüber Evidenzbasierung in ihren Organisationen, durch Gewährleistung eines regelhaften Zugangs zu Evidenzdatenbanken, durch verbesserte Kompetenzen in Bezug auf Interpretation von Evidenz und durch eine systematische Zusammenarbeit mit der Wissenschaft.

Mehr Evidenzbasierung ist eine Voraussetzung für die nachhaltige Verankerung von PGF als fünfte Säule des Gesundheitssystems.

## Abstract (en)

The German Prevention Act underlines the need for an evidence-based approach to prevention and health promotion. It is unclear which steps and processes are necessary for the evolving system of prevention and health promotion in Germany to meet this requirement. This overview article aims to define and operationalize evidence-based interventions in prevention and health promotion and describes the necessary organizational support and capacity building to foster evidence-based action in practice.

Based on the international scientific literature and the Federal Centre for Health Education’s Memorandum on Evidence-based Prevention and Health Promotion, the term evidence-based intervention is defined and operationalized, and implementation requirements in terms of organizational processes and capacity building are described and discussed.

To foster implementation of evidence-based action in practice, decision makers in policy and practice should draw on a shared understanding of the concept of evidence-based interventions and of the need for evaluations that assure generating evidence while implementing interventions. Moreover, organizational support is necessary, such as emphasizing the value of evidence-based action within organizations, ensuring access to existing evidence databases with a transparent and relevant presentation of evidence, advancing competencies of the workforce in searching for and interpreting evidence syntheses, as well as promoting a systematic cooperation between practitioners and researchers.

Implementing the above-described elements for more evidence-based action is an important step towards strengthening the evolving system of prevention and health promotion as a fifth pillar of the German health system.

## Einleitung

Prävention und Gesundheitsförderung (PGF) als Kernhandlungsfeld der Public Health hat sich in den letzten Jahren zunehmend organisiert. Angesichts der Herausforderungen des demografischen, technologischen und gesellschaftlichen Wandels und der Verschiebung der Krankheitslast hin zu chronisch degenerativen und psychischen Erkrankungen haben eine Vielzahl von AkteurInnen sowie Institutionen in Deutschland unterschiedliche Aktivitäten entwickelt. Das im Jahr 2015 verabschiedete Präventionsgesetz (PrävG) hat vonseiten des Bundes neue Impulse für die Entwicklung von PGF in Deutschland gesetzt. Das Geschehen voranbringen sollen Strukturen wie die Nationale Präventionskonferenz, die dort zu entwickelnde nationale Präventionsstrategie, der zugehörige Präventionsbericht, die dadurch geforderten kassenübergreifenden Leistungen sowie damit zusammenhängende Koordinierungsprozesse.

So hat sich ein innerhalb der zivilgesellschaftlichen, staatlich-föderalen, privaten und sozialversicherungsrechtlichen Strukturen organisiertes, sehr komplexes System der PGF herausgebildet. Zusammengenommen besteht es aus Institutionen, die teilweise in einer gewissen Hierarchie verbunden sind, aus AkteurInnen mit bestimmten Kompetenzen und Disziplinen sowie aus Regeln und Prozessen der Zusammenarbeit zwischen AkteurInnen und zwischen Institutionen. Geregelt ist diese Zusammenarbeit innerhalb des föderalen Systems mittels einer gesetzlichen Grundlage und/oder gemäß von Selbstverpflichtungen verschiedener Partner (z. B. innerhalb des Kooperationsverbundes gesundheitliche Chancengleichheit).

Die beschriebenen Charakteristika sind Kerncharakteristika eines sich organisierenden „Systems“: Systeme sind definiert als im Allgemeinen abgrenzbare, natürliche oder künstliche „Gebilde“, die aus verschiedenen Komponenten bestehen und die aufgrund bestimmter geordneter Beziehungen untereinander als gemeinsames Ganzes betrachtet werden (können; [1]). Die Organisation innerhalb eines Systems zeichnet sich aus durch Regelwerke und Instrumente zur Erreichung der Ziele, die über Organisationsmitglieder, Spielregeln, Hierarchien, Grenzen und Zwecke definiert werden [2].

Das Handeln in einem sich organisierenden System PGF in Deutschland ist wiederum in einen größeren Kontext eingebettet. Neben politischen und gesellschaftlichen Rahmenbedingungen sind dies Anforderungen, mit denen definierte „Leistungen“ im Gesundheitsbereich begründet werden müssen. Die Orientierung an der Evidenzbasierung hat beispielsweise für medizinische Leistungen der gesetzlichen Krankenversicherung Eingang in das sozialrechtliche Regelwerk und damit in die Verfahrensordnung des Gemeinsamen Bundesausschusses genommen [3]. Auch in den Bundesrahmenempfehlungen der Nationalen Präventionskonferenz sind Evidenz und Qualitätsorientierung eine wiederkehrende Terminologie [4], allerdings in abgeschwächter Form.

Somit ist Evidenzbasierung eine wichtige Anforderung an das sich entwickelnde System PGF. Evidenzbasierung in der Public Health ist definiert als: „Entscheidungen auf Grundlage einer systematischen und bewussten Integration der für die Frage relevanten besten verfügbaren wissenschaftlichen Erkenntnisse, der praktischen Erfahrungen und der Expertise relevanter Fachleute sowie der Werte und Präferenzen der betroffenen Personen“ (siehe auch Beitrag von Rehfuess et al. in diesem Themenheft und [5]).

Obwohl die Anforderung der Evidenzbasierung formuliert ist, gibt es viele Kontroversen um die Umsetzungsmöglichkeiten der Evidenzbasierung in PGF [6–8]. Die Kontroversen werden zunehmend weniger dogmatisch und die Erfahrung aus angelsächsischen Ländern zeigt, dass mehr Evidenzbasierung möglich ist und auf welche Weise sie umgesetzt werden kann. Dieser Beitrag möchte auf dieser internationalen Wissens- und Erfahrungsbasis erläutern, was die wesentlichen Elemente, Schritte, Prozesse und Vorgehensweisen für eine Evidenzbasierung und die Stärkung/den Ausbau eines Systems PGF sind.

Aufbauend auf internationalen wissenschaftlichen Erkenntnissen zu den oben genannten Themen und den Konzepten von Evidenzbasierung aus unterschiedlichen Wissenschaftsfeldern (z. B. Management, Pädagogik, Psychologie) sollen für das System PGF in Deutschland – angelehnt an das Memorandum Evidenzbasierte Prävention und Gesundheitsförderung der Bundeszentrale für gesundheitliche Aufklärung (BZgA; [9]) – folgende spezifischen Fragen beantwortet werden:

1. Wie können evidenzbasierte Interventionen der PGF operationalisiert werden als Basis für ein evidenzbasiertes Handeln von AkteurInnen in der Praxis?
2. Was sind wichtige Elemente für die Entwicklung von (organisationalen) Kapazitäten („capacity building“) für evidenzbasiertes Handeln im System PGF?

## Operationalisierung evidenzbasierter Interventionen

Es ist zu erwarten, dass ein System, das vorrangig und wo immer möglich evidenzbasierte Maßnahmen umsetzt, effektiver und effizienter ist als ein System, in dem dies nicht der Fall ist. In Deutschland ist – im Vergleich zu Ländern wie Vereinigtes Königreich [10] oder den Vereinigten Staaten [11] – die Prüfung einer Evidenzbasierung von Maßnahmen der PGF aber nicht institutionalisiert. Die Entscheidung zur Durchführung von Maßnahmen wird von unterschiedlichen AkteurInnen aus Praxis oder Politik getroffen, und zwar auf Bundes‑, Länder- und kommunaler Ebene, sowie in einzelnen Institutionen (z. B. gesetzliche Krankenkasse). Vielen dieser AkteurInnen fehlt zur Orientierung ein einheitliches Konzept der Evidenzbasierung, außerdem fehlt es an zeitlichen, personellen und finanziellen Ressourcen für die Umsetzung. Eine Grundvoraussetzung für eine Stärkung der Evidenzbasierung in PGF ist, den Begriff und die Methoden der Evidenzbasierung besser zu erklären, zu operationalisieren, Akzeptanz für seine Wichtigkeit zu fördern und bereits vorhandene Ressourcen bekannt zu machen.

### Wirksamkeit und Umsetzbarkeit evidenzbasierter Interventionen

Maßnahmen der PGF bewegen sich hinsichtlich ihrer wissenschaftlichen Absicherung innerhalb eines Spektrums, das von „deutlich und nachvollziehbar beschrieben“ über „Wirkung plausibel anzunehmen“ bis hin zu „kausale Wirkung nachgewiesen“ reicht [8].

Für evidenzbasierte Entscheidungsprozesse, welche Maßnahmen letztlich implementiert werden sollen, müssen neben Wirksamkeit auch weitere Kriterien beachtet werden. Dies sind u. a.:

- die angenommenen Wirkmechanismen von Maßnahmen (Sind die Wirkwege auch im neuen Kontext so vorstellbar?),
- die Notwendigkeit der Einbindung von Expertise und AkteurInnen aus unterschiedlichen Disziplinen (Welche spezifische Expertise wird für die Umsetzung vor Ort benötigt?),
- mögliche Wechselwirkungen von Maßnahmen im lokalen Kontext (Gibt es Merkmale, die eine Wirkung der Maßnahme erschweren oder verhindern?) sowie
- die jeweilige Akzeptanz und Machbarkeit (Erschweren lokale Gegebenheiten, dass die Maßnahme von der Zielgruppe angenommen wird?), siehe auch die Umsetzungsfaktoren nach dem Konzept TIKKA (**T**heorie, **I**nterdisziplinarität, **K**ontextabhängigkeit und **K**omplexität, allgemeine gesellschaftliche **A**spekte; [9, 12]).

Wenn jedoch in Entscheidungsprozessen die Frage aufkommt, welche evidenzbasierten Maßnahmen die Lösung für ein Problem oder einen Bedarf darstellen können, kann klar definiert werden: Die Evidenzbasierung einer einzelnen Intervention wird vor allem über ihre Wirksamkeit operationalisiert. In einem aktualisierten Konsensusstatement der American Society of Prevention Research [13, 14] werden dabei Kriterien für die Wirksamkeit unter Studienbedingungen („efficacy“, Ebene 1) und für die Wirksamkeit unter Alltagsbedingungen („effectiveness“, Ebene 2) definiert. Da in der Public Health randomisierte Evaluationen oft nur schwer oder gar nicht umsetzbar sind, werden in dem Konsensusstatement neben randomisierten Studien auch andere Studiendesigns als Voraussetzung für den Nachweis der Wirksamkeit zugelassen (z. B. unterbrochene Zeitreihenstudien oder quasiexperimentelle Designs).

Tab. 1 zeigt eine verkürzte und angepasste Zusammenfassung dieses „Standard of Evidence“ [13, 14], der zudem Kriterien für eine flächendeckende Umsetzung von Maßnahmen definiert („dissemination“, Ebene 3). Die zu erfüllenden Kriterien einer Ebene sind dabei grundsätzlich auch in allen folgenden Ebenen enthalten, d. h., die Kriterien der Ebene 1 gelten auch für die Ebenen 2 und 3. In der Praxis haben sich diese Kriterien als Instrument für eine transparente Einordnung des Wissensstands zu Maßnahmen bewiesen, so z. B. in einigen der größten Best-Practice‑/Best-Evidence-Datenbanken zu Maßnahmen der PGF in den USA („blueprints“; [15]), den Niederlanden („loketgezondleven“; [16]) und Frankreich („Portail documentaire“; [17]).

**Table 1:** 

Ebene 1	Ebene 2	Ebene 3
*Wirksamkeit unter Studienbedingungen („efficacy“)*	*Wirksamkeit unter Alltagsbedingungen („effectiveness“)*	*Wirksamkeit in unterschiedlichen Kontexten für eine flächendeckende Umsetzung („dissemination“)*
Die Maßnahme ist gut beschrieben und dokumentiert	Eine Beschreibung zur Umsetzung der Maßnahme ist vorhanden	Mindestens eine erfolgreiche Replikation in einem anderen als dem ursprünglichen Kontext war möglich
Die Zielpopulation/Studienteilnehmerinnen und -teilnehmer sind so beschrieben, dass die externe Validität beurteilt werden kann	Kernelemente der Intervention sind beschrieben oder Mediator‑/Moderatorvariablen wurden identifiziert	Umsetzungsanleitung, Trainingsunterlagen, technische Unterstützung für die Skalierung sind vorhanden, Umsetzungsvoraussetzungen bekannt
Ein logisches Modell/eine „theory of change“ ist verfügbar	Die Kontexte des alltäglichen Lebens, in denen die Intervention sich als wirksam gezeigt hat, sind beschrieben	Informationen zu Kosten der Intervention liegen vor
Für Wirkung relevante Outcomes (Endpunkte) wurden definiert, die verwendeten Outcome-Maße sind wissenschaftlich valide	Neben der Wirksamkeitsevaluation erfolgte eine Prozessevaluation zur Messung von Implementierung, Akzeptanz, Adhärenz und Partizipation	Instrumente zum Monitoring von Veränderungen bzw. Kosten durch die Maßnahme sind vorhanden
Es zeigen sich positive Effekte ohne Hinweise auf relevante unerwünschte Wirkungen, auch im Follow-up	Es wurde ein für die Praxis aussagekräftiges Outcome herangezogen bzw. Effektivität nachgewiesen	Die Monitoringstrukturen können eine gegebenenfalls notwendige Anpassung (Adaptation) der Maßnahme bei Dissemination verfolgen
Das Studiendesign lässt kausale Aussagen zu	Die Wirkung der Maßnahme wird in Abhängigkeit von der „Dosis“, die implementiert wurde, analysiert	Es gibt ein zyklisches Feedback des Monitorings zur weiteren Verbesserung/Anpassung der Maßnahme selbst oder ihrer Implementierung
Die statistische Auswertung wird dem Studiendesign gerecht, unterschiedliche Auswertungen ergeben konsistente Ergebnisse	Die Effekte können angesichts der beschriebenen Population voraussichtlich auf andere Populationen übertragen werden	
Es gibt mindestens eine Studie mit kausal aussagekräftigem Design		

### Konkrete Operationalisierung der Kriterien Wirksamkeit und Umsetzbarkeit

Laut dem kürzlich erschienenen Memorandum der BZgA [9] ist eine Maßnahme der PGF dann als evidenzbasiert zu bezeichnen, wenn sie die in Tab. 1 dargestellten Kriterien der Ebenen 1 und 2 grundsätzlich erfüllt. Mit Bezug auf die oben erwähnten TIKKA-Umsetzungsfaktoren muss für eine evidenzbasierte Intervention im Spezifischen Folgendes vorliegen:

- eine detaillierte Beschreibung der Intervention und ihrer Wirkpfade in einer bestimmten Population und einem bestimmten Kontext (logisches Modell),
- ein Nachweis ihrer Wirksamkeit und Sicherheit unter Alltagsbedingungen und
- aus Prozessevaluationen gewonnene Einsichten hinsichtlich einer erfolgreichen Umsetzung.

Da Interventionen neben positiven Effekten (= Nutzen) prinzipiell auch negative Effekte (= Schaden) haben können, ist zu fordern, dass auch potenzielle negative Effekte in einem logischen Modell verankert und als Endpunkte gemessen werden (= Nachweis von Sicherheit).

Aspekte der flächendeckenden Implementierung werden auf Ebene 3 betrachtet. Eine flächendeckende Umsetzung ist immer mit erheblichem Ressourcenaufwand und langfristigen Planungen verbunden und sollte daher noch besser abgesichert werden. Daher sollten für eine flächendeckende Umsetzung neben den Kriterien (a)–(c) weitere Kriterien erfüllt sein, nämlich:

d. dass die Wirksamkeit der Maßnahme in unterschiedlichen Kontexten gezeigt wurde,
e. dass ihre Kosten bekannt sind und erfasst werden können und
f. dass für die Umsetzung gegebenenfalls erforderliche Materialien, wie z. B. Manuale, zur Verfügung stehen (Ebene 3).

## Elemente für die Entwicklung von Kapazitäten für mehr evidenzbasiertes Handeln

Im Folgenden wird auf Basis von wissenschaftlicher Literatur und der Expertise der Autorinnen in 8 Elementen beschrieben, wie Kapazitäten für mehr evidenzbasiertes Handeln im System Prävention und Gesundheitsförderung aufgebaut werden können (Tab. 2). Die Elemente 1–4 enthalten Empfehlungen zum Verständnis und zur Anwendung des Konzeptes von evidenzbasierten Maßnahmen. Die Elemente 5–8 enthalten Empfehlungen zur Verbesserung organisationaler Rahmenbedingungen und der Kapazität von AkteurInnen und Institutionen.

**Table 2:** 

Bereich	Element	
Förderung des Verständnisses und der zielgerichteten Anwendung von evidenzbasierten Maßnahmen	1	Transparente Benennung der wissenschaftlichen Grundlage von Maßnahmen der PGF
	2	Prüfung der Transferabilität von Maßnahmen und der Notwendigkeit von Anpassungen
	3	Konsequenzen der Einordnung für die Implementierung und Evaluation von Maßnahmen
	4	Systematisches Ausschöpfen der Möglichkeiten der Wirksamkeitsevaluation von Maßnahmen
Verbesserung der organisationalen Rahmenbedingungen und der Kapazitäten von AkteurInnen und Praxisinstitutionen	5	Zugang zu und Nutzen von existierenden Datenbanken und verständliche Darstellung der wissenschaftlichen Erkenntnisse
	6	Qualifikation und Fortbildung von AkteurInnen der PGF
	7	Austausch und gemeinsames Lernen: strukturierte Rückmeldungen aus der Praxis („practice-based evidence“)
	8	Enge Vernetzung von Praxis und Wissenschaft

### Förderung des Verständnisses und der zielgerichteten Anwendung von evidenzbasierten Maßnahmen (Elemente 1–4)

Maßnahmen im System PGF in Deutschland werden oft ohne Bezug zu anderen Wissensbeständen entwickelt und z. B. als regionale oder kommunale Projekte aufgesetzt. Um die Qualität und Evidenzbasierung im Feld zu stärken, ist es daher dringend notwendig, Transparenz hinsichtlich der Verortung verschiedener Maßnahmen innerhalb des Spektrums wissenschaftlicher Absicherung herzustellen bzw. mit entsprechenden Evaluationsstrategien zur Evidenzbasierung beizutragen.

### Element 1: Transparente Benennung der wissenschaftlichen Grundlage von Maßnahmen der PGF

**Figure 1:**
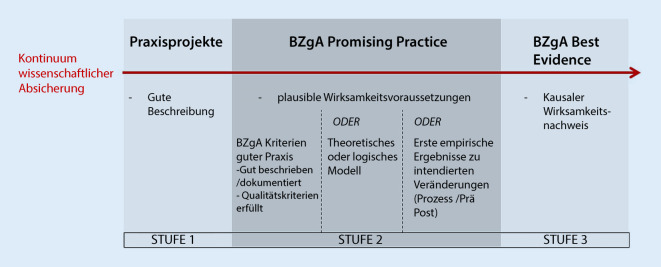

Das Memorandum der BZgA [9] schlägt für den deutschen Raum vor, die wissenschaftliche Absicherung von Maßnahmen der PGF in 2 Kategorien einzuteilen: „BZgA Promising Practice“ (BZgA vielversprechende Praxis) und „BZgA Best Evidence“ (BZgA beste Evidenz; Abb. 1). Maßnahmen, die lediglich ausreichend gut beschrieben sind, um eine Replizierung zu ermöglichen, aber sonst keine der unter „BZgA Promising Practice“ oder „BZgA Best Evidence“ genannten Kriterien erfüllen und damit auch nicht wissenschaftlich abgesichert sind, werden als Praxisprojekte bezeichnet. Sie können nur empfohlen werden, wenn Maßnahmen der anderen Kategorien in einem bestimmten Handlungsfeld nicht bestehen.

Abb. 1 zeigt die Anforderungen an die beiden oben genannten Kategorien der wissenschaftlichen Absicherung. Dabei gelten alle Kriterien in Stufe 1 auch für die Stufen 2 und 3, d. h., Maßnahmen, die als „BZgA Best Evidence“ kategorisiert werden, müssen neben einer guten Beschreibung auch Kriterien für Stufe 2 und natürlich Stufe 3 erfüllen. „BZgA-Promising-Practice“-Maßnahmen erfüllen dagegen nur die Kriterien der Stufe 2, nicht aber die der Stufe 3. Wenn mehrere Maßnahmen im Kontext akzeptabel scheinen, sollten immer Maßnahmen, die die Kriterien für „BZgA Best Evidence“ erfüllen, implementiert werden.

### Element 2: Prüfung der Transferabilität von Maßnahmen und der Notwendigkeit von Anpassungen

Vor einer Entscheidung zur Implementierung einer bestimmten evidenzbasierten Maßnahme sollte immer geprüft werden, ob diese prinzipiell in den Zielkontext übertragbar (transferierbar) ist. Welche Überlegungen hierbei leitend sein könnten und welche Fragen gestellt werden sollten, zeigt das Modell von Schloemer et al. in diesem Themenheft. Soll eine Maßnahme in einer neuen Zielpopulation oder in einem stark abweichenden Kontext zum Einsatz kommen, ist voraussichtlich eine Anpassung („adaptation“) der Intervention notwendig (siehe auch ADAPT-Guidance [18] des UK Medical Research Council oder [19]). Anpassungen können sich (a) mit dem Inhalt der Maßnahme befassen (z. B. sprachliche Anpassungen), (b) die Umsetzung der Maßnahme modifizieren (z. B. andere UmsetzungsakteurInnen) oder (c) Veränderungen im Kontext selbst vornehmen (z. B. regulatorische Änderungen, um eine Maßnahme zu ermöglichen).

### Element 3: Konsequenzen der Einordnung für die Implementierung und Evaluation von Maßnahmen

**Figure 2:**
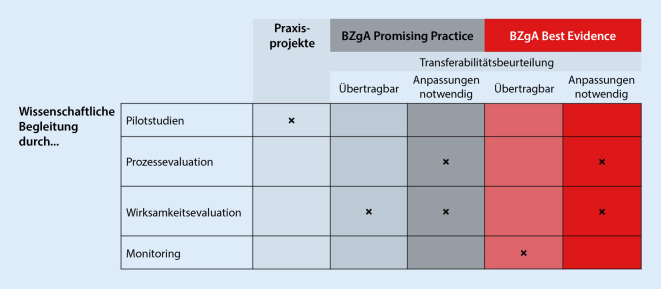

Ob und in welcher Form eine zu implementierende Maßnahme evaluiert werden muss, hängt davon ab, wo diese Maßnahme im Spektrum wissenschaftlicher Absicherung verortet ist und wie die Prüfung ihrer Transferabilität ausfällt. Handelt es sich zum Beispiel um eine „BZgA-Best-Evidence“-Intervention und wird diese als gut vom alten in den neuen Kontext übertragbar bewertet, so ist sie voraussichtlich auch im neuen Kontext wirksam. In der Konsequenz kann eine aufwendige Wirksamkeitsevaluation normalerweise entfallen ([19]; Abb. 2); eine begleitende Dokumentation von Aspekten der Implementierung und eventuellen Anpassungen der Maßnahme oder der Modalitäten ihrer Implementierung im Zeitverlauf ist jedoch in den meisten Fällen sinnvoll. Handelt es sich umgekehrt um eine „BZgA-Best-Evidence“-Intervention, die an eine neue Zielpopulation und/oder einen neuen Zielkontext angepasst werden muss (nicht direkt übertragbar), sollte mindestens eine Prozessevaluation, bei größeren Adaptationen auch eine erneute Wirksamkeitsevaluation durchgeführt werden. Eine grundsätzliche Notwendigkeit, im Ausland entwickelte und dort wirksamkeitsgeprüfte Interventionen immer auch noch einmal in Deutschland hinsichtlich ihrer Wirksamkeit zu testen, gibt es hingegen nicht. Soll allerdings eine „BZgA-Promising-Practice“-Intervention implementiert werden, dann ist es für deren Weiterentwicklung in Richtung „BZgA-Best-Evidence“-Intervention von großer Bedeutung, dass eine echte Wirksamkeitsevaluation durchgeführt wird.

### Element 4: Systematisches Ausschöpfen der Möglichkeiten der Wirksamkeitsevaluation von Maßnahmen

Eine solide Wirksamkeitsevaluation erfordert Studiendesigns, die kausale Wirkzusammenhänge möglichst eindeutig nachweisen können (siehe Kriterien der Ebene 1 und 2, Tab. 1). Prä-Post-Vergleiche von Endpunkten, die nicht unter geeigneten Studienbedingungen durchgeführt werden, aber häufig als „Evaluation“ bezeichnet werden, halten diesen Anforderungen nicht stand, sondern können nur hypothesenbildend sein. Eine Wirksamkeitsevaluation braucht immer einen geeigneten Vergleich bzw. eine Kontrolle, sei es eine Kontrollsituation, Kontrollgruppe oder eine Kontrollperiode. Auch wenn es den Idealfall darstellt, muss die Kontrolle nicht notwendigerweise randomisiert, d. h. über Zufallseinteilung bestimmt werden. Wenn eine randomisierte Zuteilung einer Maßnahme unter Alltagsbedingungen nicht möglich ist, gibt es inzwischen diverse Studiendesigns und statistische Verfahren, die trotzdem valide Erkenntnisse zur Wirksamkeit generieren können [7, 20–22]. Dazu gehören Studiendesigns wie unterbrochene Zeitreihen, bei denen die Kontrolle über einen Vergleich mit Kontrollzeiträumen in der gleichen Population vorgenommen wird, oder quasiexperimentelle Designs, in denen Kontrollsituationen mittels statistischer Methoden modelliert werden. Prozessevaluationen können im Rahmen der ersten Implementierung von Maßnahmen und Praxisprojekten wichtige Einblicke in Bezug auf die Umsetzung, Machbarkeit und Akzeptanz einer Intervention [23] bieten und sind auch nach einer eventuellen Anpassung von Maßnahmen wichtig, sie ersetzen jedoch keine Wirksamkeitsevaluation.

Aufgrund dieser komplexen Anforderung an den tatsächlichen Nachweis von Wirksamkeit ist bei der Konzipierung und Durchführung von Evaluationen eine enge Zusammenarbeit zwischen Wissenschaft und Praxis unabdingbar. Optimal ist es, die Wissenschaft schon während der Entwicklung einer Maßnahme hinzuzuziehen, damit von Anfang an eine geeignete Form der Evaluation integriert werden kann [8]. Um die Wirksamkeit von unterschiedlichen Maßnahmen im Feld Prävention und Gesundheitsförderung systematisch und nach einheitlichen Standards zu evaluieren, müssen Bedingungen erfüllt sein, die sich nicht von selbst etablieren werden. Um dies zu ermöglichen, sollte die Durchführung von aussagekräftigen Evaluationen mit Wirksamkeitsnachweis systematisch gefördert werden. Eine unterstützende Struktur, wie sie im Feld der medizinischen Versorgung zum Beispiel vom Gemeinsamen Bundesausschuss (G-BA) durch den Innovationsfonds [24] bereits seit einigen Jahren mit Erfolg bereitgestellt wird, könnte der notwendigen Entwicklung wirksamer Maßnahmen im Feld der Prävention und Gesundheitsförderung einen wichtigen Impuls geben.

### Verbesserung der organisationalen Rahmenbedingungen und der Kapazitäten von AkteurInnen und Praxisinstitutionen (Elemente 5–8)

Neben diesen Anforderungen in Bezug auf die Darstellung, Implementierung und Evaluation von Maßnahmen der PGF lassen sich auch Anforderungen für mehr evidenzbasiertes Handeln an die Institutionen im System PGF formulieren.

Als eines der umfassendsten, am besten theoretisch abgesicherten Konzepte dazu, welche Anforderungen Organisationen erfüllen müssen, um evidenzbasiert zu handeln, gilt das SPIRIT Action Framework. Es wurde im Rahmen einer großen Multikomponentenintervention entwickelt und beschreibt die Kapazität („capacity“) einer Organisation, wissenschaftliche Erkenntnisse zu nutzen, als eine Schlüsselkompetenz, um die vielbeschriebene Lücke zwischen Wissenschaft und Praxis zu schließen [25]. Die Kapazität wird an 3 Hauptkriterien festgemacht: (a) Wertschätzung von Evidenz in der Organisation (z. B. in Bezug auf die Unterstützung evidenzbasierten Handelns und die Anforderungen in Bezug auf die Nutzung von Evidenz) und durch individuelle MitarbeiterInnen, (b) Tools und Systeme, die eine Organisation für eine regelhafte Nutzung von Evidenz zur Verfügung stellt, und (c) die Kompetenzen und das Wissen der MitarbeiterInnen.

Ein Scoping-Review identifizierte basierend auf 14 systematischen Reviews und 40 Originalstudien [26] Faktoren, die eher mit evidenzbasiertem Handeln und Entscheiden in Organisationen einhergingen. Diese sind in 5 Kategorien aufgeführt: (1) Management der Integration von wissenschaftlichen Erkenntnissen in das Handeln der AkteurInnen, (2) institutionale Strukturen und Regeln für Entscheidungen, (3) Organisation und Infrastruktur für Evidenznutzung (4) organisationale Charakteristika und (5) individuelle Faktoren. Tab. 3 ist eine Übersicht der in den jeweiligen Kategorien als hochrelevant identifizierten Faktoren. Im Vergleich dieses Scoping-Reviews und des SPIRIT-Frameworks offenbaren sich viele Gemeinsamkeiten: Neben Prozessen und Standards in den Arbeitsabläufen einer Organisation sind Kultur, Werte und technische Infrastruktur wichtig. Aber auch die MitarbeiterInnen einer Organisation bedingen, insbesondere wenn sie als Führungskraft dienen, inwiefern Evidenz tatsächlich genutzt wird.

**Table 3:** 

Kategorie	1. Management der Integration von Evidenz in das Handeln	2. Institutionale Strukturen und Regeln für Entscheidungen	3. Organisation und Infrastrukturen für Evidenznutzung	4. Organisationale Charakteristika^a^	5. Individuelle Faktoren
*Hochrelevante*^*b*^* Faktoren*	Strategische Vision mit systematischer Integration von Evidenz in Praxis^c^	*Wertschätzung von Rationalität, Evaluation, Qualitätsverbesserung*	*Zugang zu Onlinedatenbanken und Repositorien*^*d*^	–	*Informelle, vertrauliche Beziehungen mit WissenschaftlerInnen*^*f*^
	Genügend Ressourcen	Hohe Wertschätzung von Evidenz in politischem Handeln	*Zielgruppenbezogene Formate der Dissemination von Evidenz*^*d*^		Empfundene Glaubwürdigkeit und Objektivität von Wissenschaft und Forschung
	Kultur, die Evidenznutzung befördert	Finanzierung von Forschung	*Persönlicher Zugang zu einer/m WissenschaftlerIn*^*f*^		*Motivation und Erwartung in Bezug auf die Nutzung von Forschung*^*c*^
	Unterstützung von Führungskräften		*Externe Partnerschaften und Kommunikationskanäle*^*e*^		Entscheidungsgewalt in der Position
	*Interne Kapazitätsentwicklung MitarbeiterInnen*^*c*^		*Interorganisationale Netzwerke gemeinsamen Lernens*^*e*^		Hohe Bildung
	In Bezug auf Evidenz ansprechbare Mitarbeitende		Unterstützung durch Bibliotheksdienste oder Informationsspezialisten		Vorhandensein interner Experten
					Vorbilder für die Nutzung von Evidenz
					*Kompetenz in der Suche und Interpretation von Forschungsergebnissen, eigene Forschungserfahrung*^*d*^

^a^In der Kategorie 4 konnten keine hochrelevanten Faktoren identifiziert werden

^b^Hochrelevant: mit Nennung in mindestens 7 Studien im Scoping-Review (mind. 1 x in einem Review)

Kursiv markiert sind solche Faktoren, die in diesem Artikel in die konzipierten Elemente für mehr evidenzbasiertes Handeln einfließen. Hochgestellte Buchstaben bezeichnen, in welche Elemente die Faktoren einfließen:

^c^Grundlage für Elemente 1–4

^d^Grundlage für Elemente 5–6

^e^Grundlage für Element 7

^f^Grundlage für Element 8

### Element 5: Zugang zu und Nutzen von existierenden Datenbanken und verständliche Darstellung der wissenschaftlichen Erkenntnisse

Es existieren bereits diverse Datenbanken, in denen systematische Übersichtsarbeiten sowie Evidenz zur Wirksamkeit von Maßnahmen und zu ihren Wirkvoraussetzungen im internationalen Raum gesammelt und präsentiert werden. Alle Organisationen, die in der PGF arbeiten, sollten diese Datenbanken und Repositorien kennen, im Sinne eines Wissensmanagements an ihre MitarbeiterInnen weitergeben und den Zugang dazu technisch sichern, z. B. durch Kooperationen mit Universitäten. Die wichtigsten Datenbanken für systematische Übersichtsarbeiten sind in Infobox 1 dargestellt.

Oftmals sind die primären Nutzenden WissenschaftlerInnen, weshalb manche Datenbanken teils (noch) nicht genügend auf die Bedürfnisse der NutzerInnen aus der Praxis eingehen. Einige große Datenbanken stellen Informationen allerdings schon durch neue, besser für die Praxis zugängliche Formate zur Verfügung, z. B. als laienverständliche Zusammenfassungen auf Englisch oder Deutsch. Vereinfachte Formate der Kommunikation von Erkenntnissen aus systematischen Übersichtsarbeiten werden entwickelt [27]. Bewährt hat sich dabei eine mehrschichtige Darstellung mit (a) Hauptbotschaften, (b) Zusammenfassung und (c) Detailbeschreibung [28, 29]. Für den deutschsprachigen Raum könnte in Zukunft auch die Datenbank „Wissen für gesunde Lebenswelten“ des Bündnisses für Gesundheit der Gesetzlichen Krankenversicherungen (GKV; [30]) eine Rolle spielen. Insgesamt lässt sich feststellen, dass die praxisrelevanten Ergebnisse aus systematischen Übersichtsarbeiten in den letzten Jahren deutlich zunehmen, aber sicherlich noch mehr Evidenz durch Interventionsstudien generiert und über geeignete praxis- und politikfreundliche Formate der Aufbereitung vermittelt werden muss [16], damit PGF in der Fläche tatsächlich evidenzbasiert umgesetzt werden kann.

Neben Datenbanken für systematische Übersichtsarbeiten gibt es auch Datenbanken, die transparent das bestehende Wissen zu einzelnen Maßnahmen darstellen. Für eine vollständige Übersicht verweisen wir auf Tab. 6 im Memorandum der BZgA [9]. Der Vorteil dieser Datenbanken ist, dass sie einzelne Maßnahmen auf die Bedarfe und Kompetenzen der NutzerInnen bezogen vorstellen, diese hinsichtlich ihrer wissenschaftlichen Absicherung transparent kategorisieren und auch AnsprechpartnerInnen angeben, die zu allen Themen der Umsetzung einer Maßnahme kontaktiert werden können. Dies befördert das gemeinsame Lernen und den interorganisationalen Austausch, ein hochrelevanter Faktor im Scoping-Review von Jakobson et al. ([26]; Tab. 3).

### Element 6: Qualifikation und Fortbildung von AkteurInnen der PGF

Kompetenzen von MitarbeiterInnen in Organisationen sind von entscheidender Bedeutung (Tab. 3) für mehr evidenzbasiertes Handeln. Der Wert, den Institutionen dem Konzept der Evidenzbasierung beimessen, muss daher gestärkt werden, z. B. durch eine offene und explizite Haltung und ein gemeinsames Verständnis von Führungskräften oder durch eine klare Kommunikation dazu, dass systematische Übersichtsarbeiten eine effiziente Möglichkeit der Information sind, da sie basierend auf soliden Methoden einen schnellen Überblick sowie qualitätsgesicherte Interpretation aller für eine festgelegte Fragestellung relevanten Originalstudien ermöglichen [29].

Auf der Ebene der MitarbeiterInnen ist eine Reihe praktischer Kompetenzen und Fähigkeiten notwendig: Darunter fallen die Kompetenz, mittels Schlagworte zielführend und effizient zu suchen, Fachbegriffe im Englischen zu verstehen, existierende Datenbanken und Ressourcen zu kennen, systematische Übersichtsarbeiten verstehen und interpretieren zu können und Evaluationen zum richtigen Zeitpunkt in Kooperation mit der Wissenschaft zu planen. Diese Kompetenzen müssen bei allen AkteurInnen im Feld systematisch gestärkt werden. Hierzu braucht es einen Kompetenzrahmen für in der PGF Tätige und dazugehörige Fortbildungen mit einheitlichen Standards über das Bundesgebiet hinweg [29]. Für den erfolgreichen und nachhaltigen Aufbau eines solchen Qualifizierungssystems ist der partizipative Einbezug von professionellen Institutionen und Netzwerken (z. B. Landesvereinigungen für Gesundheit, Öffentlicher Gesundheitsdienst, Kooperationsverbund gesundheitliche Chancengleichheit, Fachgesellschaften) unabdingbar.

### Element 7: Austausch und gemeinsames Lernen: strukturierte Rückmeldungen aus der Praxis („practice-based evidence“)

Empfehlungen, Evidenzsynthesen und Maßnahmen, die in Lebenswelten implementiert werden sollen, müssen praxistauglich beschrieben, relevant und für die NutzerInnen umsetzbar sein. Deshalb sollten genau diese Aspekte rückgemeldet werden können, z. B. im Rahmen von Datenbanken, die mit den NutzerInnen interagieren oder durch nachgeschaltete Befragungen. So schlagen auch Trojan und Kolip vor, dass die Gemeinschaft der GesundheitsförderInnen z. B. bestehende Projektdateien in der Datenbank des Kooperationsverbunds gesundheitliche Chancengleichheit ergänzen sollte, um Praxiserfahrungen in Bezug auf Gelingensfaktoren, Barrieren und weitere Bewertungen einfließen zu lassen [16]. So könnte die in der internationalen Literatur diskutierte „practice-based evidence“ tatsächlich auch die Evidenzbasierung des gesamten Feldes der PGF voranbringen und damit den empirisch gesicherten Faktor „interorganisationale Netzwerke gemeinsamen Lernens“ (Tab. 3) aufgreifen.

### Element 8: Enge Vernetzung von Praxis und Wissenschaft

Kooperationen zwischen Wissenschaft und Praxis sind notwendig, um den Nachweis von Wirksamkeit und die wissenschaftliche Betrachtung anderer Kriterien für die PGF zu erbringen. Vertraute Beziehungen mit WissenschaftlerInnen sind dementsprechend ein empirisch gesicherter, hochrelevanter Faktor für mehr evidenzbasiertes Handeln in Praxisorganisationen (siehe die mit hochgestelltem Buchstaben f gekennzeichneten Faktoren in Tab. 3). Dies bedeutet konkret, dass WissenschaftlerInnen frühzeitig (1) bei in der Praxis anstehenden Entscheidungen systematisch und regelmäßig, z. B. über Gremien, mit einbezogen werden. Zudem sollten sie (2) bei der Auswahl existierender Interventionen bzw. der Entwicklung neuer Interventionen und (3) bei der Prozess- und Wirksamkeitsevaluation von Interventionen der PGF beteiligt werden. Grundlage für wissenschaftliche Beratung sollte hierbei die Methodik der Entwicklung, Umsetzung und Evaluation von komplexen Interventionen in der Public Health sein, wie beschrieben in den Guidance- Dokumenten des UK Medical Research Council [31]. In diese Richtung geht z. B. das Angebot des GKV-Bündnisses für Gesundheit, Krankenkassen bei der externen Evaluation von krankenkassenartenübergreifenden Maßnahmen der Gesundheitsförderung und Prävention in Lebenswelten zu unterstützen [32].

## Fazit

Dieser Übersichtsartikel skizziert angelehnt an das Memorandum der BZgA [9], welches Grundverständnis von Evidenzbasierung bei der Umsetzung von Maßnahmen der Prävention und Gesundheitsförderung notwendig ist und wie dieses im Handeln von einzelnen AkteurInnen und von Organisationen im System operationalisiert werden kann.

Neben der transparenten und einheitlichen Darstellung in Datenbanken und Empfehlungen ist es notwendig, bei EntscheidungsträgerInnen in Praxis und Politik ein gemeinsames Verständnis von evidenzbasierten Interventionen und von Anforderungen für eine Evaluation, die Evidenzbasierung sichert, zu schaffen. Mit einer entsprechenden Evaluationsstrategie kann auch die Implementierung von Maßnahmen mit geringer wissenschaftlicher Absicherung, aber möglicherweise hohem Wirkungspotenzial zur Evidenzbasierung beitragen.

Zudem ist es in Bezug auf organisationale Rahmenbedingungen und Kapazitäten von entscheidender Bedeutung, die Wertschätzung von Evidenzbasierung in Praxisorganisationen insbesondere bei Führungskräften zu fördern, MitarbeiterInnen einen gesicherten Zugang zu Evidenzdatenbanken zu ermöglichen, ihre Kompetenzen und ihr Wissen nachhaltig durch Qualifizierung zu stärken und die Wissenschaft frühzeitig und systematisch in Entscheidungen zu Maßnahmen der PGF einzubeziehen.

Die Berücksichtigung der im Text skizzierten 8 Elemente ist ein Schritt auf dem Weg zu mehr Evidenzbasierung als Voraussetzung dafür, Prävention und Gesundheitsförderung nachhaltig als fünfte Säule des Gesundheitssystems neben Diagnostik, Therapie, Pflege und Rehabilitation zu etablieren.

### Infobox 1 Übersicht über Datenbanken zu Evidenzsynthesen, gemäß [9]

- Cochrane Library (https://www.cochranelibrary.com) – Cochrane-Reviews und laienverständliche Kurzzusammenfassungen auf Englisch
- Cochrane Kompakt (https://www.cochrane.org/de/evidence) – laienverständliche Kurzzusammenfassungen einer Vielzahl von Cochrane-Reviews auf Deutsch
- Campbell Collaboration (https://campbellcollaboration.org/) – Campbell-Reviews zu Fragen aus den Bereichen Soziales und Bildung
- Epistemonikos (https://www.epistemonikos.org/) – Datenbank systematischer Übersichtsarbeiten zu gesundheitlichen Fragestellungen hinsichtlich Wirksamkeit und einer Vielzahl anderer Fragestellungen in 9 Sprachen, darunter Englisch und Deutsch

## References

[1] Willemse J, Von Ameln F (2018). Theorie und Praxis des systemischen Ansatzes. Die Systemtheorie Watzlawicks und Luhmanns verständlich erklärt.

[2] Ansmann L, Baumann W, Gostomzyk J (2019). DNVF-Memorandum III – Methoden für die Versorgungsforschung, Teil 4 – Konzept und Methoden der organisationsbezogenen Versorgungsforschung. Kapitel 1 – Definition und Konzept der organisationsbezogenen Versorgungsforschung. Gesundheitswesen.

[3] Gibis B, Klakow-Franck R, Schlottmann N, Bruns J, Kunz R, Ollenschläger G, Raspe H, Jonitz G, Donner-Banzhoff N (2007). Systemsteuerung im Rahmen des SGB V: der Gemeinsame Bundesausschuss. Lehrbuch evidenzbasierte Medizin in Klinik und Praxis mit 85 Tabellen.

[4] Nationalen Präventionskonferenz (Npk) (2018). Bundesrahmenempfehlungen nach § 20d Abs. 3 SGB V. Erste weiterentwickelte Fassung vom 29. August 2018.

[5] Von Philipsborn P, Rehfuess E, Schmidt-Semisch H, Schorb F (2021). Evidenzbasierte Public Health. Public Health: Disziplin – Praxis – Politik.

[6] Borrmann S, Thiessen B (2016). Wirkungen Sozialer Arbeit. Potenziale und Grenzen der Evidenzbasierung für Profession und Disziplin.

[7] Gerhardus A, Rehfuess E, Zeeb H (2015). Evidence-based health promotion and prevention in settings: which types of study designs are needed?. Z Evid Fortbild Qual Gesundhwes.

[8] Veerman JW, Van Yperen TA (2007). Degrees of freedom and degrees of certainty: a developmental model for the establishment of evidence-based youth care. Eval Program Plann.

[9] De Bock F, Dietrich M, Rehfuess E (2020). Evidenzbasierte Prävention und Gesundheitsförderung.Memorandum der Bundeszentrale für gesundheitliche Aufklärung (BZgA).

[10] National Institute for Health and Care Excellence (Nice) (2021) What we do. https://www.nice.org.uk/about/what-we-do. Zugegriffen: 23. März 2021

[11] The Community Guide (2021) About the community preventive services task force. https://www.thecommunityguide.org/task-force/about-community-preventive-services-task-force. Zugegriffen: 23. März 2021

[12] Rehfuess E (2021) Internationales Verständnis von Evidenz-basierung in Prävention und Gesundheitsförderung – Eine Übersicht und Analyse bestehender Definitionen. Bundesgesundheitsblatt Gesundheitsforschung Gesundheitsschutz : (Schwerpunktheft Mai 2021. Evidenzbasierung in Prävention und Gesundheitsförderung)

[13] Flay BR, Biglan A, Boruch RF (2005). Standards of evidence: criteria for efficacy, effectiveness and dissemination. Prev Sci.

[14] Gottfredson DC, Cook TD, Gardner FE (2015). Standards of evidence for efficacy, effectiveness, and scale-up research in prevention science: next generation. Prev Sci.

[15] University of Colorado Boulder (Institute of Behavioral Science) (2021) The blueprints for healthy youth development. https://www.blueprintsprograms.org/. Zugegriffen: 23. März 2021

[16] Rijksinstituut Voor Volksgezondheid En Milieu Ministerie Van Volksgezondheid (2021) https://www.loketgezondleven.nl/. Zugegriffen: 23. März 2021

[17] Sante Publique France (2021) Répertoire des interventions efficaces ou prometteuses en prévention et promotion de la santé. https://www.santepubliquefrance.fr/a-propos/services/interventions-probantes-ou-prometteuses-en-prevention-et-promotion-de-la-sante/repertoire-des-interventions-efficaces-ou-prometteuses-en-prevention-et-promotion-de-la-sante. Zugegriffen: 23. März 2021

[18] Moore G, Copeland L, Craig P et al (2021) Adaptation of interventions for implementation and/or re-evaluation in new contexts: The ADAPTguidance (v1.0). https://decipher.uk.net/wp-content/uploads/2020/11/ADAPT-guidance-for-website-upload-v1.0-12-10-20.pdf. Zugegriffen: 23. März 2021

[19] Evans RE, Craig P, Hoddinott P (2019). When and how do ‘effective’ interventions need to be adapted and/or re-evaluated in new contexts? The need for guidance. J Epidemiol Community Health.

[20] Craig P, Cooper C, Gunnell D (2012). Using natural experiments to evaluate population health interventions: new Medical Research Council guidance. J Epidemiol Community Health.

[21] Craig P, Katikireddi SV, Leyland A, Popham F (2017). Natural experiments: an overview of methods, approaches, and contributions to public health intervention research. Annu Rev Public Health.

[22] Trojan A, Kolip P (2020). Evidenzbasierung in der Prävention und Gesundheitsförderung.

[23] Moore GF, Audrey S, Barker M (2015). Process evaluation of complex interventions: medical research council guidance. BMJ.

[24] Gemeinsame Bundesausschuss (2021) Förderbekanntmachungen. https://innovationsfonds.g-ba.de/foerderbekanntmachungen/. Zugegriffen: 23. März 2021

[25] Redman S, Turner T, Davies H (2015). The SPIRIT Action Framework: a structured approach to selecting and testing strategies to increase the use of research in policy. Soc Sci Med.

[26] Jakobsen MW, Eklund Karlsson L, Skovgaard T, Aro AR (2019). Organisational factors that facilitate research use in public health policy-making: a scoping review. Health Res Policy Syst.

[27] Busert LK, Mutsch M, Kien C (2018). Facilitating evidence uptake: development and user testing of a systematic review summary format to inform public health decision-making in German-speaking countries. Health Res Policy Syst.

[28] Lavis J, Davies H, Oxman A, Denis JL, Golden-Biddle K, Ferlie E (2005). Towards systematic reviews that inform health care management and policy-making. J Health Serv Res Policy.

[29] Wallace J, Byrne C, Clarke M (2012). Making evidence more wanted: a systematic review of facilitators to enhance the uptake of evidence from systematic reviews and meta-analyses. Int J Evid Based Healthc.

[30] GKV-Spitzenverband (2021) GKV-Bündnis für Gesundheit. Datenbank „Wissen für gesunde Lebenswelten. https://www.gkv-buendnis.de/forschung-im-buendnis/datenbank-wissen-fuer-gesunde-lebenswelten/. Zugegriffen: 23. März 2021

[31] Medical Research Council (2021) Leading science for better health. https://mrc.ukri.org/. Zugegriffen: 23. März 2021

[32] GKV-Spitzenverband (2021) Unterstützung der Krankenkassen bei Evaluationen. https://www.gkv-buendnis.de/forschung-im-buendnis/unterstuetzung-bei-evaluationen/. Zugegriffen: 23. März 2021

